# Design and Characterization of High-Performance Borophene Oxide-Reinforced PLA/PEG Composite Films

**DOI:** 10.1021/acsomega.5c13427

**Published:** 2026-03-13

**Authors:** Merve Ercan Kalkan, Sema Samatya Yilmaz, Nurseli Görener Erdem, Murat Efgan Ki̇bar, Esra Bi̇lgi̇n Şi̇mşek, Ayşe Aytaç

**Affiliations:** † Department of Chemical Engineering, Engineering Faculty, 52980 Kocaeli University, Kocaeli 41380, Türkiye; ‡ Polymer Science and Technology, Institute of Science, Kocaeli University, Kocaeli 41380, Türkiye; § Department of Chemical Engineering, Engineering Faculty, Gebze Technical University, Gebze, Kocaeli 41400, Türkiye

## Abstract

Borophene oxide (BO) was synthesized using a modified Hummer method and added at different ratios into a plasticized polylactic acid/polyethylene glycol (PLA/PEG) matrix via a solution-casting technique. Characterization of the produced films for potential packaging applications was carried out through thermal, mechanical, structural, morphological, and antibacterial tests. The addition of 0.5 wt % BO to the plasticized PLA film resulted in an approximately 72% increase in the tensile strength (22.6 MPa). Increasing the BO content decreased the elongation values. In TGA analysis, the Td_10_ temperature of the PLA/PEG matrix increased from 310 °C to approximately 338 °C with the addition of a low amount of BO. The water vapor barrier of the plasticized PLA films was enhanced with 0.5–0.7 wt % BO addition but diminished at higher BO concentrations. The resulting films exhibited greater antibacterial activity against *S. aureus* than *E. coli*. Considering all film properties, the developed films were shown to be promising for biomedical and especially packaging applications, where the vapor permeation barrier and Gram-positive bacterial contamination are critical concerns due to the BO content.

## Graphical abstract

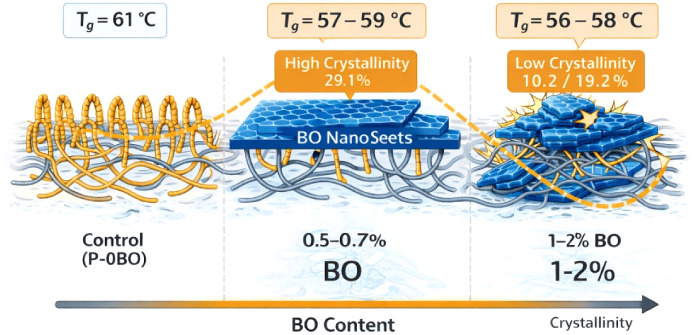

## 1 Introduction

Boron is a versatile element that has attracted significant attention in materials science and nanotechnology due to its unique properties.^1^ Its derivatives exhibit a wide range of functional characteristics, making them suitable for various implementations. Hexagonal boron nitride (h-BN) is a nonmetallic material with high thermal conductivity and a large surface area. It is also an excellent electrical insulator, offering robust resistance to electrical currents. These properties make h-BN a potential material for photocatalytic and electronic applications.^2,3^ On the other hand, borophene, a two-dimensional form of boron, offers metallic conductivity, mechanical flexibility, and a unique structure that enables tunable electronic properties. These characteristics make it a promising material for advanced electronic and energy-related applications.^4,5^ However, its low stability prevents borophene from being used on a large scale.^6^ Borophene oxide (BO), an oxidized form of borophene, provides improved chemical stability. This enhancement makes BO a promising candidate for use in broad applications.^6,7^ Studying boron and its derivatives is important for developing new functional materials. The unique properties of boron-based materials offer opportunities for advancements in various fields, including electronics, energy storage, and composite systems. Boron, an essential trace element, has attracted increasing interest in biomedical applications due to its involvement in bone metabolism, cell proliferation, and wound healing. Although elemental boron is not used directly as an implant material, boron-containing biomaterials such as borate-based bioactive glasses, boron-doped hydroxyapatite, and composite scaffolds have demonstrated good biocompatibility and enhanced osteogenic activity when applied at controlled concentrations. Previous studies have reported that the controlled release of boron ions can stimulate osteoblast differentiation and accelerate tissue regeneration; however, excessive boron concentrations may induce cytotoxic effects. Therefore, the safety and efficacy of boron in biomedical applications are strongly dependent on its chemical form, dosage, and release kinetics.^8–11^

Recent review studies on two-dimensional boron, commonly referred to as borophene, provide comprehensive insights into its synthesis, structural characteristics, and potential applications. Borophene synthesis has been extensively reviewed through a variety of approaches including chemical vapor deposition (CVD), molecular beam epitaxy (MBE), and liquid-phase sonochemical exfoliation, which influence the resulting polymorphic borophene structures and their properties such as electronic conductivity, mechanical flexibility, and anisotropic behavior.^5,12,13^ These studies also discuss the emerging potential of borophene for applications in sensing, energy devices, and advanced functional materials, highlighting ongoing efforts to stabilize and exploit boron-based 2D materials.^5,13^

Polylactic acid (PLA) is widely used because it is biodegradable, biocompatible, and derived from renewable resources. However, pure PLA is brittle, has low thermal resistance, and weak barrier properties.^14,15^ Adding boron-based nanofillers to PLA has been suggested to improve these limitations. Even small amounts of boron derivatives can enhance PLA’s mechanical and thermal performance. Boron nitride has been frequently used as an additive in many studies. Bindhu et al. developed PLA-based composites incorporating 0–4 wt % boron nitride (BN) through a solvent casting technique.^16^ SEM analysis showed BN aggregation at the highest loading. Among the samples, the 2 wt % BN composites showed the strongest tensile performance, achieving an increase of about 132% over neat PLA. Qin et al. prepared PLA/boron nitride nanotube composites by melt mixing.^17^ Their results showed that a small amount of BN improved PLA crystallization. The crystallization mechanism remained unchanged, indicating that BN acts as an effective nucleator. Guo et al. conducted a study on PLA/boron nitride composite scaffolds prepared via 3D printing.^18^ They found that adding boron nitride improved mechanical strength, biomineralization, and cell response. Optimal mechanical properties were observed at 1 wt % boron nitride, while higher amounts caused aggregation. Boron nitride also promoted apatite formation and supported stem cell growth and bone differentiation. Ara et al. prepared PLA composite films reinforced with micro- and nanosized boron nitride.^19^ The films contained 1–10 wt % boron nitride and were tested for physical, mechanical, and barrier properties. Tensile strength improved by 28% with 1 wt % microsized boron nitride and by 40% with 1 wt % nanosized boron nitride, while water vapor permeability was significantly reduced. However, the films exhibited no antimicrobial activity. Muhammed et al. prepared PLA films with hexagonal boron nitride (hBN) and hBN-ZnO nanofillers.^20^ Films with 1.5 wt % hBN showed the best performance: tensile strength increased, water vapor transmission decreased, and UV and heat resistance improved. The addition of ZnO gave antibacterial, antibiofilm, and antioxidant properties, while mechanical and barrier properties were preserved. Yin et al. prepared PLA/PEG-based phase change composites with boron nitride (BN) by melt processing.^21^ BN improved thermal conductivity, shape stability, and antileakage performance. The phase change enthalpy remained nearly the same, while thermal stability improved, with the decomposition temperature rising by almost 50 °C.

Recent studies have highlighted the development of polymer and hybrid nanofiller composites for sustainable applications, particularly in food packaging. For instance, poly­(lactic acid) reinforced with nanocellulose has been reported to exhibit enhanced tensile strength, improved thermal stability, and reduced water vapor permeability.^22^ In addition, comprehensive studies and edited volumes by Jacob and coworkers emphasize that filler type, morphology, and dispersion state play a critical role in governing the mechanical, thermal, and barrier performance of polymer-based composites.^23,24^ These insights provide a relevant framework for the exploration of boron-based nanofillers, which may be incorporated within similar composite design strategies to further enhance the functional performance of PLA films while introducing additional properties. Avcı et al. studied PLA composites with different boron-based fillers, including zinc borate, boric acid, borax, and ulexite.^25^ Boric acid and borax slowed down PLA combustion, improving fire resistance. All boron fillers increased flexural and Young’s modulus, while tensile strength slightly decreased. The thermal degradation temperature was slightly reduced by the addition of boron compounds. The best mechanical and thermal performance was observed for composites containing 5 wt % boron fillers. Kangallı and Bayraktar prepared PLA/boron oxide nanoparticle (BONPs) composites using solution casting.^26^ The BONPs were reduced in size and modified with oleic acid, methacrylate, and silane-based additives. Adding these nanoparticles improved the thermal, mechanical, and flame-resistant properties of PLA. The composites showed higher crystallinity, stronger tensile strength, and better flame resistance.

Several studies have investigated borophene-reinforced polymer nanocomposites. Das et al. produced borophene and polyvinylidene fluoride (PVDF) nanofibers for triboelectric nanogenerators.^27^ Borophene was synthesized using a sonochemical method, and the fibers were formed by electrospinning. The nanogenerators generated high electrical output and could power small devices. Single-electrode devices efficiently captured energy from raindrops. Banerjee et al. investigated epoxy composites with a few-layer borophene. Mechanical properties improved, with higher stiffness and lower thermal expansion.^28^ Three to four borophene layers gave the best reinforcement. Adekoya et al. investigated a polymer blend of 3,4-ethylenedioxythiophene and polystyrenesulfonate containing borophene nanoplatelets.^29^ They used finite element simulations to examine the composites. The results showed that adding borophene increased the elastic modulus of the films. Türkmen et al. prepared poly­(3,4-ethylenedioxythiophene):polystyrenesulfonate nanocomposites incorporated with β12 borophene. Borophene with a β12 crystalline phase was synthesized using sonication, and its addition enhanced the electrodes’ capacitance.^30^

In our previous study, the plasticized PLA films containing boron carbon nitride (BCN) were prepared by using the solution casting method. BCN was added at 0.5–2 wt %, and the films were tested for mechanical, thermal, chemical, and structural properties. Tensile strength improved at 1 wt % BCN, and thermal stability increased with BCN addition. Crystallinity remained mostly unchanged, while water wettability was enhanced. Antibacterial tests showed stronger effects against *Staphylococcus aureus* than *Escherichia coli*.^15^ To the best of our knowledge, the fabrication and characterization of polylactic acid (PLA) films incorporating borophene oxide have not yet been reported in the literature. In this study, borophene oxide was synthesized in our laboratory and incorporated in varying amounts into plasticized PLA with PEG. The resulting composites were characterized for their structural, thermal, mechanical, morphological, barrier, and antibacterial properties.

## 2 Materials and Methods

### 2.1 Materials

Polylactic acid (PLA, 4043D) was obtained from NatureWorks LLC, and polyethylene glycol (PEG, P2139) was purchased from Sigma-Aldrich. Chloroform (CF) solvent was supplied by Carlo Erba Reagents. Boron precursor obtained from Pavezyum Kimya PavTec, Türkiye.

### 2.2 Methods

#### 2.2.1 Synthesis of BO

BO was synthesized using a modified Hummer method.^31–34^ Initially, 100 mg of pure boron powder was mixed with potassium permanganate (KMnO_4_) at a weight ratio of 1:6, followed by grinding for 10 min. An acidic mixture was prepared by mixing ortho-phosphoric acid (H_3_PO_4_) and sulfuric acid (H_2_SO_4_) at a ratio of 1:9 (v:v). Since the reaction was exothermic, a precooling procedure was applied by cooling these two mixtures below 5 °C for 6 h. The boron powder + KMnO_4_ mixture was added to the acidic mixture and subjected to ultrasonication (Scientz JY92-IN) at 65 °C for 10 min. Then, 200 mL of distilled water was added to terminate the reaction, and the mixture was ultrasonicated for another 10 min. After the reaction was completed, the resulting solid was washed three times with distilled water (200 mL) and twice with ethanol (50 mL) and dried at 65 °C.

#### 2.2.2 BO-Added Plasticized PLA Films Preparation

As the first step in solution preparation, PLA was dried in a vacuum oven at 80 °C overnight. Subsequently, a 5% (w/v) PLA solution was prepared in chloroform, and PEG was added at a PEG:PLA weight ratio of 1:5. BO was then incorporated at four different concentrations corresponding to 0.5, 0.7, 1, and 2 wt % of PLA. The solutions were mixed using a mechanical stirrer for 18 h and then homogenized with a homogenizer. Film formation from the prepared solutions was carried out as described in our previous work.^15^ Film thickness was carefully controlled using identical Petri dishes and the same casting volume, and all measurements were averaged over at least three independent specimens.

The sample without BO was designated as the “control” and labeled as “P”. The prepared films were named according to their BO content. For example, the film P-1BO represents PLA films plasticized with PEG containing 1% BO by weight. Table 1 provides an overview of the labeling and composition of the prepared films.

**Table 1:** Composition of Prepared Films

Sample Code	P	P-0.5BO	P-0.7BO	P-1BO	P-2BO
PLA % (by weight)	5	5	5	5	5
PEG/PLA ratio (by weight)	1:5	1:5	1:5	1:5	1:5
BO (wt % of PLA)	-	0.5	0.7	1	2

### 2.3 Characterization

The structural and chemical features of composite films were examined using Fourier-transform infrared spectroscopy (FTIR). Spectral data were collected on a PerkinElmer Spectrum 100 instrument (PerkinElmer, Waltham, MA, USA) across a 4000–500 cm^–1^ range with four scans per sample. Through this analysis, the functional groups were identified, and the molecular interactions between the polymer chains and BO nanosheets were clarified.

The structure of BO was examined from 10° to 80° with a step size of 0.02 using CuKα radiation (λ = 0.15418 nm) at 45 kV/40 mA for X-ray diffraction (XRD) (Rigaku, Miniflex 2). The crystal structures of the composites were investigated by X-ray powder diffraction (XRD, Rigaku D-Max 2200) with Cu Kα radiation scanned from 10° to 45° 2θ with a scanning rate of 10° min^–1^.

Mechanical properties were assessed through uniaxial tensile testing on an Instron universal testing machine following ASTM D882 standards. Five replicates per film were analyzed to report average tensile values.

Thermal stability was investigated using thermogravimetric analysis (TGA) on a Mettler Toledo TGA 1 system. Samples were heated from 25 °C to 600 °C at a rate of 10 °C/min under nitrogen flow (30 mL/min).

Differential scanning calorimetry (DSC) was performed using a Mettler Toledo DSC1 Star System in the temperature range of 25–300 °C under a nitrogen atmosphere, with a heating rate of 10 °C/min and conducted as a single run.

Surface morphology was visualized using a Philips XL30 SFEG scanning electron microscope (SEM), while elemental composition was evaluated with Energy Dispersive X-ray Analysis (EDX) spectroscopy integrated into the SEM. The sheet-like structure and the thickness of B and BO were characterized on 300-mesh carbon coated copper grids with scanning transmission electron microscopy (STEM) operated at a 30.00 kV accelerating voltage using a Quattro S from Thermo Fisher Scientific.

Barrier properties were assessed through water vapor transmission rate (WVTR) tests. WVTR was measured on a Mocon Permatran-W 101 K system in line with ASTM D6701, with a measurement range of 500–100,000 g/(m^2^·day) and three replicates per sample.

Inductively coupled plasma–optical emission spectroscopy (ICP-OES) analysis was performed using Optima 7000 DV (PerkinElmer, USA) to evaluate the leaching behavior of B element. Zeta potential of each sample was measured using Malvern Zetasizer Nano-ZS to assess the effect of BO loading on the mechanical properties of P-BO films.

#### 2.3.1 Antibacterial Test

Gram-positive *Staphylococcus aureus* *(* *S. aureus* *)* and Gram-negative *Escherichia coli* *(* *E. coli* *)*, stored at −80 °C in glycerol stocks, were used as model microorganisms. Briefly, 5 μL aliquots of each bacterial stock were streaked separately on Luria–Bertani (LB) agar plates (BioShop) using sterile disposable inoculating loops. The plates were incubated at 37 °C overnight. A single colony from each culture was subsequently transferred into 5 mL of sterile LB broth (BioShop) and incubated at 37 °C with agitation overnight to obtain fresh inocula. These liquid cultures, derived from single colonies, were used for subsequent antibacterial assays with film samples. To evaluate the effect of the samples on bacterial growth in liquid media, each film sample (100 mg) was added to 20 mL LB broth inoculated with 1 mL of bacterial suspension (OD600 ≈ 1; ∼8 × 10^8^ CFU/mL). Control cultures containing only bacteria (without film samples) were prepared for both *S. aureus* and *E. coli*. All cultures were incubated at 37 °C in a shaking incubator at 100 rpm. Absorbance values at 600 nm were recorded at defined time intervals using a spectrophotometer.^35^ After 4 h of incubation, OD600 values were compared between the sample-containing and control groups to assess growth inhibition. Following the incubation period, liquid cultures were diluted 1:1000 with fresh LB broth. Aliquots of 100 μL were spread on LB agar plates and incubated overnight (16–18 h) at 37 °C. Colony-forming units (CFUs) were then counted to evaluate the effect of the samples on bacterial viability, and the results were compared with the control groups.

#### 2.3.2 Disk Diffusion Test

Film samples were cut into uniform-sized disks (7 mm in diameter) and sterilized under ultraviolet (UV) light for 20 min. As positive and negative controls, sterile disks impregnated with 10 μL ciprofloxacin (0.1 mg/mL) and 10 μL LB broth, respectively, were employed. Fresh bacterial suspensions were prepared by adjusting the optical density at 600 nm (OD600) to 1.0, corresponding to approximately 8 × 10^8^ CFU/mL.^36^ A 100 μL aliquot of each bacterial suspension was evenly spread onto LB agar plates. The sterilized sample disks, together with the positive and negative control disks, were placed on the agar surface (two sample disks and one control disk of each type per plate) and incubated at 37 °C for 16–18 h. After incubation, inhibition zones were identified as transparent regions without bacterial growth around the disks. Zone diameters were recorded from digital images using ImageJ software.

## 3 Results and Discussion

### 3.1 FTIR Analysis

Structural characteristics of the samples were examined using FTIR spectroscopy. FTIR spectra are presented in Figure 1. According to Figure 1a, the control sample exhibited characteristic peaks in the ranges of 2940–3000 cm^–1^ and around 1747 cm^–1^, as well as at approximately 1450 cm^–1^, 1380–1350 cm^–1^, 1186 cm^–1^, 1080 cm^–1^, 868 cm^–1^, and 752 cm^–1^. The absorptions observed between 2940 and 3000 cm^–1^, along with those near 1450 and 1380–1350 cm^–1^, can be attributed to the C–H stretching and bending vibrations of the CH_3_ and CH groups of PLA.^37,38^ The prominent peak at 1747 cm^–1^ corresponds to the C=O stretching vibration of ester groups, which is characteristic of PLA. Additionally, the bands at 1186 cm^–1^ and 1080 cm^–1^ are associated with C–O and C–O–C stretching vibrations, which can be assigned to both PLA and PEG segments.^37,39^ The weaker peaks observed around 868 and 752 cm^–1^ are ascribed to CH out-of-plane bending and CH_2_ rocking modes, which are typically related to the semicrystalline domains of PLA or to conformational motions within PEG chains.^40^

**Figure 1:**
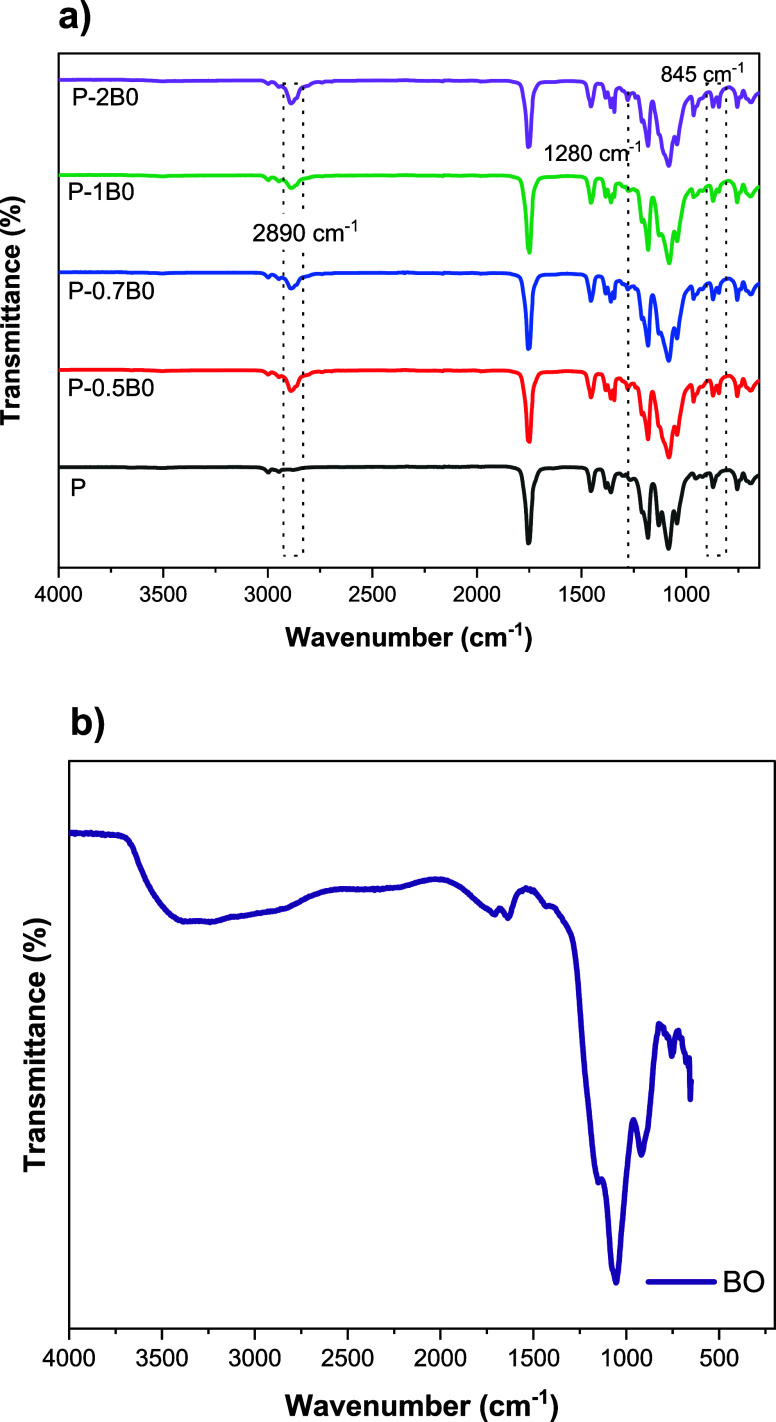
FTIR spectra showing the vibrational characteristics of the samples (a) FTIR spectra of the film samples (b) FTIR spectrum of BO.

Figure 1b shows the FTIR spectrum BO, exhibiting characteristic vibrational bands reflecting its structural motifs. A broad band at 3400–3200 cm^–1^ corresponds to O–H stretching vibrations, indicative of hydroxyl groups or adsorbed moisture. The peaks at 1712 and 1627 cm^–1^ are associated with C=O and C=C stretching, likely due to minor surface-adsorbed species. Strong bands at 1156 and 1051 cm^–1^ correspond to B–O stretching in trigonal BO_3_ and tetrahedral BO_4_ units, while the bands at 915, 747, and 656 cm^–1^ are attributed to B–O–B bending vibrations. These spectral features confirm the characteristic vibrational modes of BO and provide insights into its structural arrangement.^41–43^

The FTIR spectra of the BO-containing films exhibited distinct bands at approximately 2890 cm^–1^, 1280 cm^–1^, and 845 cm^–1^, which were absent in the control sample P. The band at 1280 cm^–1^ is attributed to the asymmetric stretching of B–O bonds in trigonal BO_3_ units, while the 845 cm^–1^ band corresponds to stretching vibrations of tetrahedral BO_4_ units and bending modes of bridging oxygen (B–O–B) between BO_3_ and BO_4_ units, in agreement with previous studies on boron-containing materials. The band at 2890 cm^–1^ is likely associated with C–H stretching vibrations influenced by interactions between the polymer chains and incorporated BO. The presence of these bands confirms the formation of boron-rich networks within the polymer matrix, reflecting the coexistence of both BO_3_ and BO_4_ structural units.^41,42,44^ These spectral features indicate that BO is effectively incorporated into the polymer matrix, leading to structural modifications and potentially altering the composite’s physicochemical properties.

### 3.2 XRD Analysis

Figure 2a shows the XRD diffractograms of bulk boron as precursor and the prepared BO by modified Hummer’s method. The prepared BO and the boron exhibit β-rhombohedral crystalline structure where both had the same peaks at 11.2, 17.6, 19.0 and 20.1° correspond to the (003), (104), (021), and (015), respectively.^45–47^

**Figure 2:**
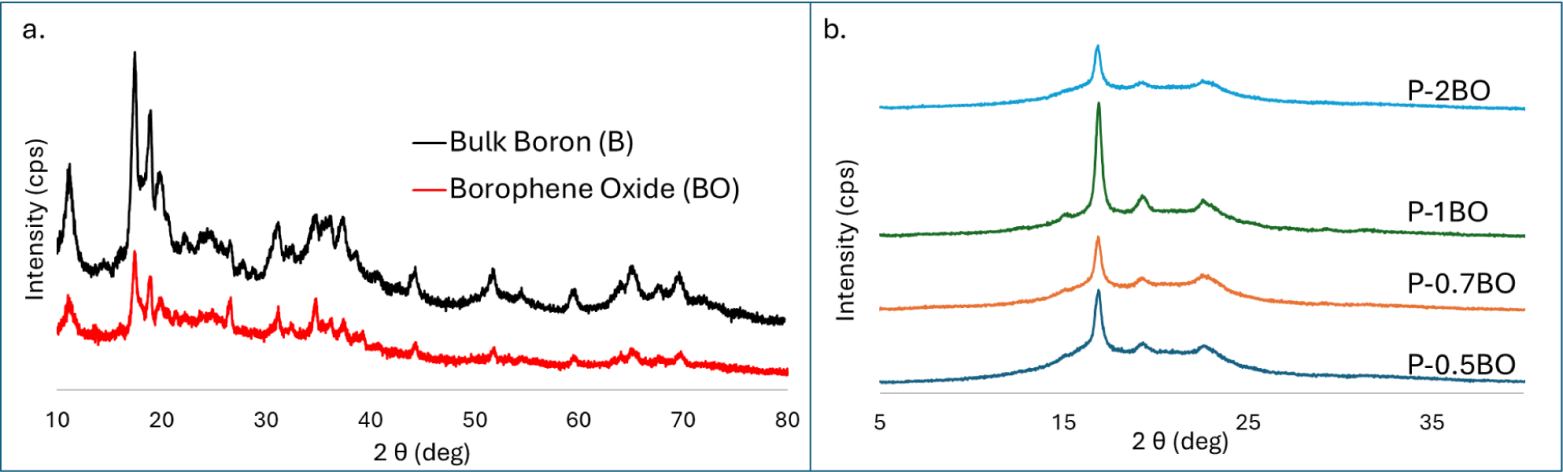
XRD spectra of (a) bulk boron and BO, and (b) BO-loaded films.

In Figure 2b, the spectra of the films are given according to different BO loading amounts. Characteristic peaks of PEG at 19° and 22.7° correspond to the (115) and (016) planes, respectively. The peak at 16.9° is attributed to PLA.^48^ According to Figure 2b, the peak intensity of PEG is not affected by BO addition, but the intensity of PLA decreases up to 1.0 wt % loading of BO, then dramatically increases. This indicates that agglomeration or heterogeneous distribution of BO occurred in the PLA matrix. This result confirms the FTIR results that there is a decrease in intensity for 1.0 wt % loading at the 2890 cm^–1^ band. Under these heterogeneous conditions, the limited interaction between BO and PEG affects the mechanical properties of the PLA/PEG composites.

### 3.3 TGA and DSC Analysis

The thermal property data of the films are summarized in Table 2. When evaluated together, the TGA and DTG curves presented in Figure 3, along with the DSC thermograms shown in Figure 4, collectively demonstrate that the incorporation of BO significantly influences the thermal behavior and enhances overall stability of the 80PLA/20PEG matrix.

**Table 2:** Data on Thermal Properties of Films

Samples	*T*_deg10_ (°C)	*T*_max_ (°C)	Residue at 600 °C (%)	*T*_g_ (**°**C)	*T*_c_ (°C)	*T*_m_ (°C)	Crystallinity (%)
**P-0.5BO**	337	364	0.91	57	99	156	29.1
**P-0.7BO**	340	366	0.90	59	99	156	29.1
**P-1BO**	339	365	1.17	58	99	156	10.2
**P-2BO**	337	365	1.53	56	100	156	19.2

**Figure 3:**
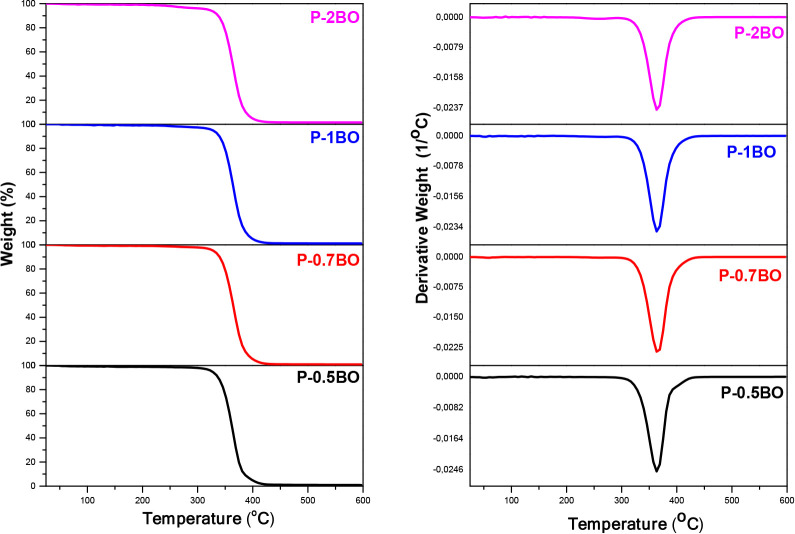
TGA and DTG curves of BO-added films.

**Figure 4:**
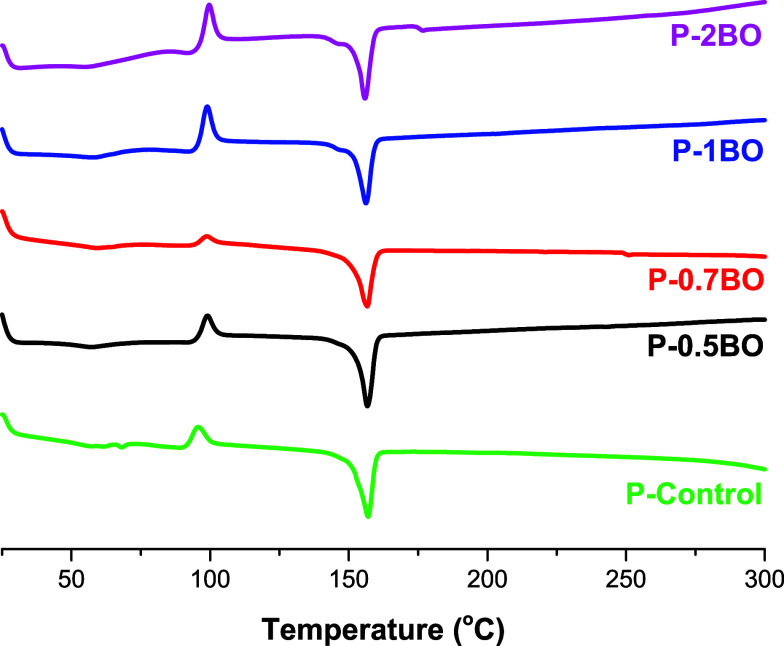
DSC curves of BO-added films.

In our previous study, the P-Control sample exhibited two distinct degradation stages at temperature values of 348 °C for *T*_max1_ and 396 °C for *T*_max2_, corresponding to the degradation of PLA and PEG phases, respectively. Additionally, the *T*_deg10_ temperature, corresponding to a 10% mass loss, was determined to be 310 °C.^15^ In contrast, the *T*_max1_ and *T*_max2_ degradation temperatures in films modified with 0.5, 0.7, 1, and 2% BO additions showed a single and unified *T*_max_ degradation temperature at approximately 365 °C (±2). This reveals the presence of strong interfacial interactions between the PLA/PEG matrix components and BO. The transition from two-stage to single-stage degradation indicates that BO molecules containing reactive −OH groups form B–O–C bonds with the hydroxyl ends of PEG chains or the ester groups of PLA. These bonds increase phase compatibility and limit independent phase separation.^49^ BO-added samples increased the thermal stability of the PLA/PEG matrix, showing a 10% weight loss at approximately 338 °C (±2), an increase of approximately 9% compared to the control sample.

The amount of residue measured at 600 °C was 1.29% in the P-Control sample, while it was found as 0.90, 0.91, 1.17, and 1.53% for the films containing 0.5%, 0.7%, 1%, and 2% BO, respectively. The P-0.5BO and P-0.7BO samples exhibited nearly identical residue amounts, suggesting that at low and closely spaced BO concentrations, comparable residual mass values may occur, which can be attributed to the possible agglomeration of the additive in certain regions of the film from which the test specimens were taken. The decrease in residue amounts at low BO addition rates (0.5, 0.7, and 1%) is attributed to the evaporation of water released because of condensation reactions between the −OH groups of BO and the PEG or PLA chain ends. This suggests the formation of covalent or semicovalent B–O–C linkages within the matrix, leading to reinforced interfacial linkage.^49^ As the BO addition rate increases (2%), the active binding sites in the matrix reach saturation, leaving unreacted BO species or self-condensed BO structures, which result in the formation of thermally stable inorganic residues after degradation. Therefore, at lower BO concentrations, bond formation via condensation predominated, while at higher BO concentrations, increased residue formation occurred due to saturation. This bidirectional effect demonstrates that BO functions as both a compatibilizer and a thermal stabilizer, highlighting the importance of optimizing the additive concentration to achieve maximum thermal stability.

The *T*_g_ and *T*_m_ of the pure PLA film with 5% concentration were recorded as 61 and 157 °C, respectively. The *T*_m_ of the PEG powder was found to be 64 °C.^50^ The thermal transitions observed in the P-Control film obtained in this study corresponded to the temperature values of 57 and 157 °C for *T*_g_ for PLA, and *T*_m_ for PLA, respectively. After the mixture of PLA and PEG, a decrease in the *T*_g_ of PLA was observed, and this result was associated with the plasticizing effect of PEG on PLA. With the decrease in *T*_g_, chain movements increased, and the elongation at break behavior of the pure PLA film was improved.^50^ Additionally, according to the literature, the *T*_c_ temperature of PLA is around 110 °C,^51^ while the crystallization temperature of PEG varies depending on its molecular weight, particularly in the 25–40 °C range.^52,53^ A single crystallization temperature (*T*_c_) of 96 °C was observed in the P-Control film. This study reported that the presence of PEG slightly reduced the crystallization temperature of PLA.^54^ The *T*_g_ and *T*_c_ temperatures of the BO-added films were found to be similar to those of the P-Control film, and it was reported that the presence or increasing amounts of BO did not notably affect the *T*_g_ or *T*_c_ of the films. The *T*_g_ and *T*_c_ temperature of all films was found to be 57 °C (±2) and approximately 99 °C, respectively. With the addition of BO, a dominant single melting point (*T*_m_) around 156 °C (±0.5) was observed. Since single *T*_g_ points are characteristic of compatible polymer blends, it can be concluded that BO is compatible with PLA and PEG and does not cause phase separation.^55^

The degree of crystallinity (*X*_c_) of the P-Control film, calculated relative to PLA, increased from 25.9%^15^ to approximately 29% in the P-0.5BO and P-0.7BO films. The increased degree of crystallinity indicated that BO had a nucleating effect, facilitating PLA chain alignment. Further increases in the BO addition amount (≥1%) resulted in a decrease in the *X*_c_ of the films (10.2–19.2%). This result is attributed to the strong localized B–O–C interactions and enhanced interfacial bonding that restrict the chain mobility required for crystal growth.

Overall, TGA and DSC analyses confirm that an optimal BO content (0.5–0.7%) enhances the thermal resistance, crystallinity, and interfacial compatibility of the PLA/PEG matrix through specific chemical interactions. These findings indicate that boron-based compounds can effectively enhance the thermal stability and crystallization behavior of polymeric materials.

### 3.4 Mechanical Test Results

Table 3 presents the mechanical properties obtained from tensile tests, including tensile strength, modulus, elongation at maximum load, and elongation at break. Borophene acted as a reinforcing agent in samples with 0.5%, 0.7%, and 1% content, improving tensile strength. The highest tensile strength increase, about 72% was observed at 0.5% borophene. However, further increases in BO content reduced this improvement. As the borophene content increased to 0.7% and 1%, the mechanical properties slightly decreased compared to the 0.5% sample but remained higher than that of the reference film. This is likely due to minor agglomeration or less uniform dispersion at higher loadings, which reduces reinforcement efficiency. At 2% borophene content, the mechanical properties drop slightly below the reference. This decrease may result from significant particle agglomeration and poor dispersion, which create stress concentration points and weaken the composite.^56^ Thus, while low borophene concentrations enhance mechanical performance, excessive addition can negatively affect the material, indicating an optimal reinforcement level around 0.5% to 1%. In our study, Young’s modulus and tensile strength changed in parallel, indicating that the additives increased both the stiffness and strength of the films.^57^

**Table 3:** Mechanical Properties of Films

Sample	Tensile strength (MPa)	Young’s modulus (MPa)	Elongation at maximum load (%)	Elongation at break (%)	Film thickness (μm)
**P**	13.11 ± 1.62	1229 ± 28	3.12 ± 0.48	29.97 ± 9.97	98±3
**P-0.5BO**	22.60 ± 1.20	1457 ± 47	2.46 ± 0.16	4.07 ± 0.46	
**P-0.7BO**	18.87 ± 0.75	1378 ± 68	2.40 ± 0.42	2.74 ± 0.54	
**P-1BO**	18.10 ± 0.67	1262 ± 83	2.10 ± 0.18	2.68 ± 0.29	
**P-2BO**	12.50 ± 0.91	1117 ± 82	1.48 ± 0.21	1.80 ± 0.40	

Since PLA is brittle, films were formulated with the plasticizer PEG as mentioned before. While the addition of borophene increased tensile strength, it caused a notable decrease in elongation at maximum load and elongation at break. This suggests that borophene reduces the plasticizing effect of PEG in the matrix. This is likely due to interactions between PEG and borophene. The combined effect of PEG and borophene strengthened the matrix and increased tensile strength but lowered ductility, making the material more brittle. This may be caused by restricted chain movement due to additive interactions, as evidenced by the FTIR spectrum.^58–60^ As a result, PEG’s ductility improvement could not balance borophene’s stiffening effect, producing a stronger but less flexible material. The decrease in elongation with higher borophene content supports this finding.

### 3.5 SEM and EDX Analysis

SEM micrographs of the BO powder and BO-loaded PLA/PEG films (P-BO) are shown in Figure 5. The BO powder exhibits a crumpled, wrinkled, sheet-like morphology that forms cauliflower-like secondary agglomerates. Such multilayer, corrugated platelets are favorable for mechanical interlocking with polymer matrices. For the BO-loaded films, each composition is presented as a fracture cross-section (50 μm scale bar) and a surface view. The SEM images of the P-0.5BO sample indicate that the cross-section is continuous and relatively compact with a tortuous fracture path and no obvious interfacial voids, suggesting effective wetting and adhesion between BO and the PLA/PEG matrix. The top surface is largely smooth with subtle submicron corrugations; occasionally, bright microislands are consistent with well-dispersed BO platelets at or near the surface. On the other hand, P-0.7BO shows a dense, uniform cross-section; however, its surface is smoother than P-0.5BO and exhibits fewer protrusions/bright features. At this magnification, no discrete BO clusters are evident, suggesting lower surface exposure of the filler. The SEM images of P-1BO composite reveal pronounced surface wrinkling with a ridge–valley topography.

**Figure 5:**
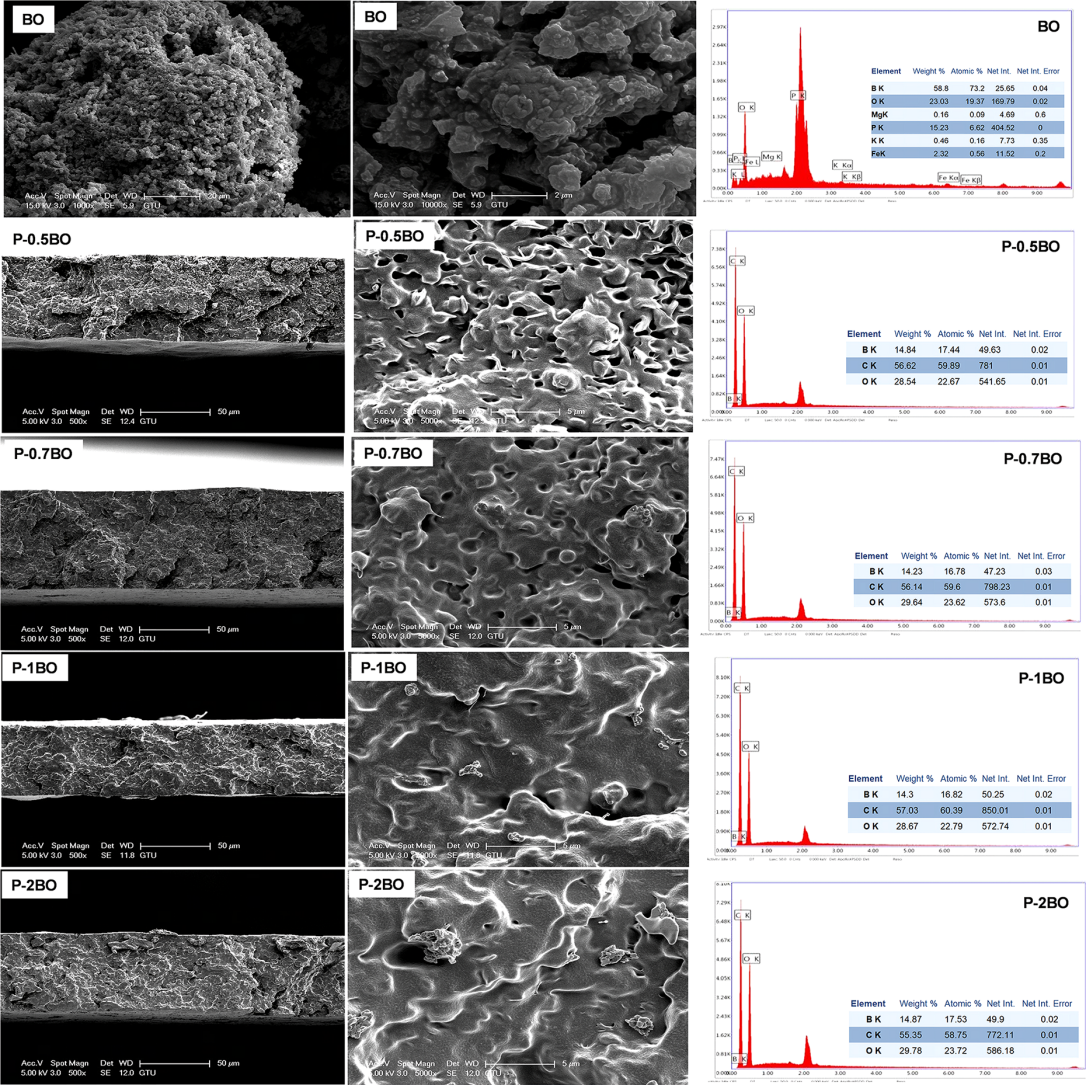
SEM images and EDX analysis of BO powder and P-BO composite films with increasing BO ratios.

The fracture cross-section displays a more tortuous crack path with occasional microvoids, consistent with higher filler loading and the onset of microagglomeration. The bright surface “islands” are more numerous than in P-0.5BO/P-0.7BO, indicating greater BO exposure at the film–air interface. After increasing BO loading, the cross-section shows discernible filler aggregates embedded in the matrix, and the top surface displays pronounced ridges and several micron-scale clusters, consistent with local BO enrichment. The combination of increased roughness and exposed BO domains provides a plausible microstructural basis for the strongest antibacterial activity observed for this formulation. Overall, BO addition increases surface microtexture and, at ≥1 wt.%, yields visible BO-rich domains. These features can enhance bacteria–surface contact and/or facilitate local BO availability, cohering with the larger inhibition zones measured for the higher-loaded films in the disk-diffusion assay.

The SEM images showed that at low borophene (BO) content (0.5%), stress was efficiently transferred to the matrix due to the smooth morphology and effective filler–matrix interaction, which was directly correlated with the highest observed increase in tensile strength (approximately 72%). When the BO content was increased to 0.7% and 1%, increased surface roughness and microagglomerations partially disrupted the filler distribution, thereby reducing the reinforcement efficiency. This explained the limited increase in tensile strength and elastic modulus compared to the sample containing 0.5% BO. At the highest BO content (2%), significant agglomeration and irregular distribution led to stress concentrations, which were associated with a decrease in mechanical properties. In conclusion, the SEM findings demonstrated that effective mechanical reinforcement was achieved at low BO contents, while agglomeration limited mechanical performance at higher contents.

To determine the sheet-like structure and the thickness, the STEM images of B and BO were given in Figure 6. The STEM images of B and BO were obtained for bright, dark and high-angle annular dark-field (HAADF) with different magnifications. The average thicknesses of B and BO were determined as 7.9148 and 7.9150 nm, respectively. These results confirm that the exfoliation has been successfully achieved. While these results appear to be consistent with the literature,^47^ it has also been concluded that modified Hummers’ preparation method is a powerful method for consistently forming homogeneous sheet structures.

**Figure 6:**
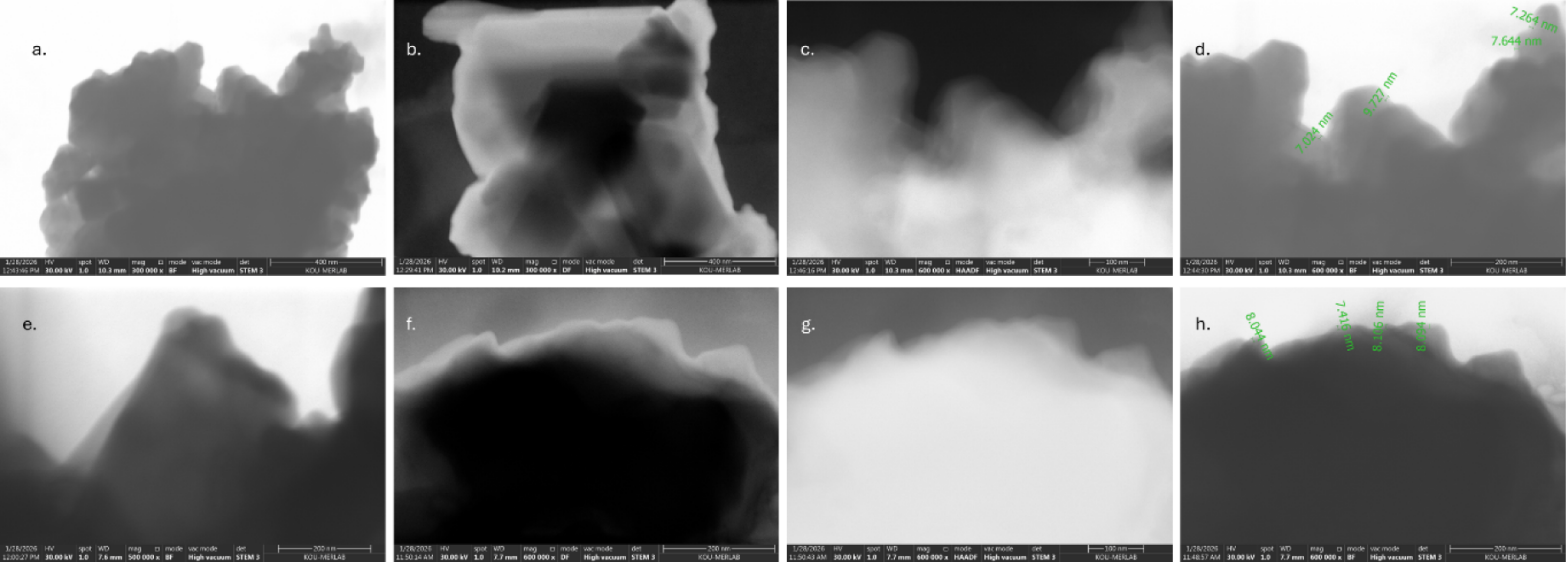
STEM images with different magnification of B (a–d) and BO (e–h).

### 3.6 Zeta Potential Analysis

To further clarify the agglomeration mechanism, zeta potentials of each sample was measured at constant pH. Zeta potential is a significant indicator of dispersion stability, reflecting the magnitude of electrostatic repulsion between dispersed species. When the zeta potential closes to zero, electrostatic stabilization weakens and attractive forces become dominant leading to particle agglomeration.^61^ As demonstrated in Figure 7, zeta potential was measured as 2.75 mV at the lowest BO loading (P-0.5BO). However, it markedly decreased from 2.75 mV (P-0.5BO) to 0.10 mV (P-2BO) as BO ratio increased within the PLA matrix, indicating a progressive loss of electrostatic stabilization with increasing BO content. Therefore, weakened dispersion stability could promote the agglomeration of BO particles within the PLA matrix at higher loadings, yielding clustered morphologies rather than a uniform distribution. Such agglomeration could lead to structural heterogeneities that act as stress concentration sites and generate interfacial defects resulting in deteriorated mechanical integrity and barrier performance.^62^ Consequently, zeta potential results confirmed the insufficient electrostatic stabilization at higher BO loadings within a PLA matrix leading to aggregation-driven microstructural defects.

**Figure 7:**
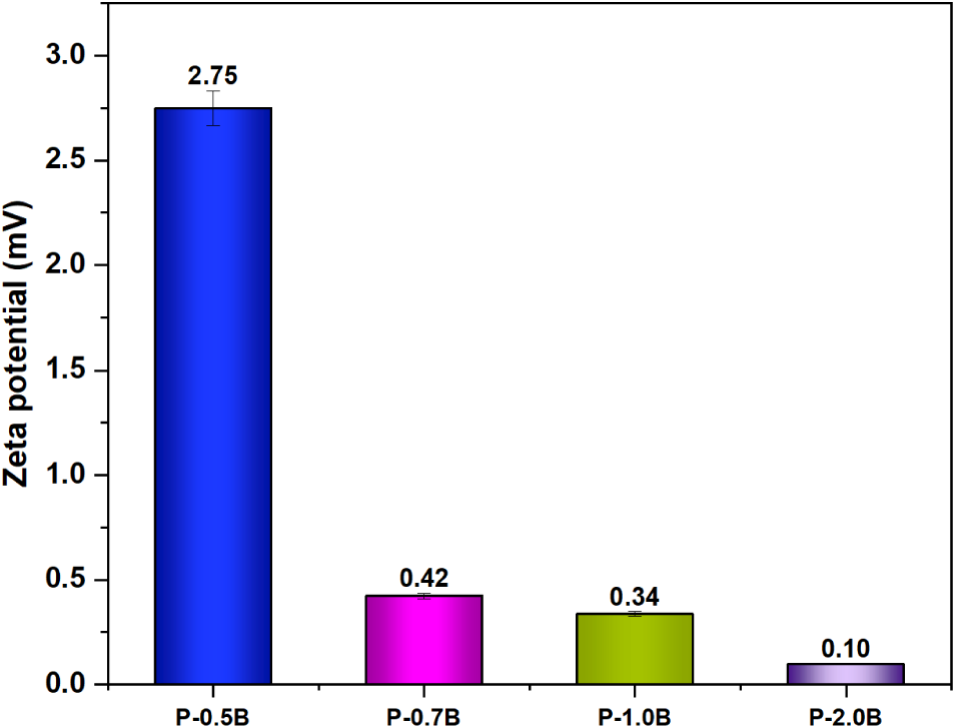
Zeta potential measurements of as-fabricated P-BO films.

### 3.7 Water Vapor Permeability Test Results

The results obtained from the water vapor transmission rate (WVTR) measurements are provided in Table 4. WVTR measurements reveal the effect of BO incorporation on the barrier properties of PLA/PEG films. The control film called P, exhibited a relatively high WVTR of 505.42 g/m^2^/day, indicating limited resistance to water vapor permeation. Introducing BO at low concentrations (0.5 and 0.7 wt %) resulted in a substantial reduction in WVTR, reaching 293.33 and 254.16 g/m^2^/day, respectively. This reduction suggests that BO contributes to improved polymer chain organization, forming a denser matrix that effectively restricts water vapor diffusion.^63,64^ However, further increasing the BO content to 1 and 2 wt % led to elevated WVTR values of 310.5 and 354.67 g/m^2^/day, implying that excessive additives may disrupt film uniformity, potentially due to agglomeration or phase separation, thereby reducing barrier efficiency.^65,66^ These findings are also consistent with the mechanical properties. The results highlight the significance of optimizing BO concentration, with 0.7 representing an optimal level that balances structural integrity and water vapor resistance, thereby achieving the most effective enhancement of barrier performance.

**Table 4:** WVTR Results of Films

Sample Code	WVTR (g/(m^2^·day))
P	505.42
P-0.5BO	293.33
P-0.7BO	254.16
P-1BO	310.50
P-2BO	354.67

Water vapor permeation in PLA-based films is governed by polymer chain mobility, crystalline–amorphous structure, film morphology, and environmental conditions. Previous studies have shown that variations in crystallinity and molecular mobility strongly influence sorption and diffusion processes in PLA, thereby affecting overall permeability. The incorporation of nanofillers further modifies transport behavior by increasing diffusion tortuosity and introducing interfacial effects, as well as by altering crystalline morphology and polymer–filler interactions. Accordingly, barrier performance in PLA systems has been interpreted using different modeling concepts depending on filler geometry, dispersion state, and the validity of underlying assumptions, rather than through a single universal model.^22,63,67,68^

At low to moderate BO contents, the reduction in water vapor transmission observed in this study can be rationalized by an increased diffusion path length and enhanced polymer–filler interactions, consistent with the qualitative framework of Nielsen-type tortuosity models commonly applied to PLA nanocomposites containing high aspect ratio and two-dimensional fillers. At higher BO loadings, the partial loss of barrier performance suggests increasing microstructural heterogeneity, likely associated with filler agglomeration and local disruption of film continuity, as widely reported for PLA-based nanocomposites. In this context, the applied modeling framework is employed to rationalize the experimental permeation behavior and observed trends, rather than as a fully predictive quantitative model.

### 3.8 Antibacterial Tests

#### 3.8.1 Disk Diffusion Test

The antibacterial performance of the composite films was evaluated using the disk diffusion method, and the inhibition zones formed around the samples are presented in Table 5. The inhibition diameters were calculated by subtracting the original disk diameter (7 mm) from the total clear zone diameters observed after incubation. Crucially, increasing the Borophene loading correlated with larger inhibition zones: P-2BO produced 3.79 mm (*S. aureus*) and 2.59 mm (*E. coli*), P-1BO yielded 2.49 mm and 1.77 mm, respectively, while P-0.5BO showed only limited activity (<1 mm). Relative to P-2BO, P-1BO was 34.3% less effective against *S. aureus* and 31.7% less effective against *E. coli*; P-0.5BO was 77.3% and 67.2% less effective, respectively. This dose–response increase with boron content mirrors the broader literature on boron-containing fillers in polymers, where introducing boron nanoparticles into PVA nanofibers significantly enlarged zones of inhibition versus neat PVA, and broad-spectrum activity was confirmed by disk diffusion assays.^69^ Similarly, boron nitride nanosheets and boron-modified systems have shown intrinsic bacteriostatic/antibacterial behavior and synergistic improvements in polymer matrices, including reports of BN-based hybrids enhancing antibacterial performance and BN flakes exhibiting antibacterial effects when oriented within composites.^70^ Finally, within a closely related PLA/PEG platform, boron-containing BCN fillers increased inhibition areas, and the enhanced activity—particularly against *S. aureus*—was attributed to boron’s antimicrobial contribution. On the other hand, it was evident that *E. coli* demonstrated higher resistance to all film samples than *S. aureus*, which is consistent with the intrinsic differences between Gram-negative and Gram-positive bacterial cell wall structures.

**Table 5:** Inhibition Zones (mm Formed around the Film Samples against *S. aureus* and *E. coli* ^a^

Inhibition zone (mm)	*S. aureus*	*E. coli*
Positive control (Ciprofloxacin, 0.1 mg/mL)	79.65 ± 3.64	79.91 ± 4.50
P-2BO	3.79 ± 0.65	2.59 ± 1.35
P-1BO	2.49 ± 0.91	1.77 ± 0.05
P-0.5BO	0.86 ± 1.20	0.85 ± 0.38
Negative control (LB broth-impregnated disk)	0	0

a Data are presented as the mean ± standard deviation of two independent biological replicates

#### 3.8.2 Antibacterial Activity in Liquid Culture

To evaluate the effects of the films on bacterial proliferation, absorbance values (OD600) of the control and sample-containing bacterial cultures were recorded immediately after the samples were introduced and again after 4 h of incubation. At this point, the experiment was terminated, and the final OD600 readings were obtained. The results are presented in Table 6. According to the results, when the growth of control cultures was set as 100% at 4 h, *E. coli* and *S. aureus* reached 94.1% and 73.3% growth, respectively, in the presence of P-2BO. In cultures containing P-1BO, *E. coli* exhibited 125% growth (indicating an enhancement compared to control), while *S. aureus* reached 79.4% growth. The P-0.5BO film reduced bacterial proliferation, resulting in 85.3% growth for *E. coli* and 72.7% for *S. aureus*. These findings demonstrate that, among the tested films, P-2BO and P-0.5BO inhibited the growth of *E. coli*, whereas *S. aureus* growth was inhibited by P-2BO, P-1BO, and P-0.5BO. In the agar-based disk diffusion assay, inhibition zones increased with borophene loading (P-0.5BO < P-1BO < P-2BO), indicating a dose-dependent enhancement of antibacterial activity. By contrast, in liquid culture the response was not strictly monotonic—at 4 h, P-0.5BO reduced growth more than P-2BO—suggesting medium-dependent diffusion/release kinetics and/or dispersion–aggregation effects at higher borophene loadings. Taken together, the data support that introducing borophene strengthens the antibacterial performance of the films, with the magnitude of the dose effect depending on the assay conditions. This dose-dependent trend is consistent with recent polymer studies where adding an additional antimicrobial to the matrix enlarged disk-diffusion zones.^71^

**Table 6:** Absorbance Values (OD600) of Liquid Bacterial Cultures (*S. aureus* and *E. coli*) Containing Different Films and Their Relative Growth Percentages Compared to Control Cultures (Set as 100%)

	Abs_*t*=0 min_		Abs_*t*=240 min_		Growth %	
Sample	*E. coli*	*S. aureus*	*E. coli*	*S. aureus*	*E. coli*	*S. aureus*
Control	0.05	0.049	1	1.253	100	100
P-2BO	0.05	0.049	0.941	0.919	94.1	73.3
P-1BO	0.05	0.049	1.258	0.996	125	79.4
P-0.5BO	0.05	0.049	0.853	0.912	85.3	72.7

To further evaluate the antibacterial properties of the films, bacterial colony formation was examined on LB agar plates. For *S. aureus*, cultures grown in the presence of the films produced noticeably fewer colonies compared to the control group when spread on LB agar (Figure 8). These results corroborate the findings of the disk diffusion and liquid culture assays, confirming the antibacterial activity of the films. Moreover, the colony count analysis demonstrated that *S. aureus* was more susceptible to the films than *E. coli*, highlighting the differential sensitivity of Gram-positive and Gram-negative bacteria to the tested samples. The results demonstrated that the BO included in PLA/PEG films exhibited greater antimicrobial efficacy against *S. aureus* than against *E. coli*. This observation suggested that the tested materials were more effective against Gram-positive bacteria, a trend commonly reported in the literature.^72^ The differences could be explained by the structural characteristics of the bacterial cell envelope. While *S. aureus* contains a thick peptidoglycan layer that is directly exposed to the external environment, *E. coli* possesses an additional outer membrane composed of lipopolysaccharides. This outer membrane functions as a selective barrier that restricts the passage of many antimicrobial agents, thereby conferring higher intrinsic resistance to Gram-negative bacteria.^73^

**Figure 8:**
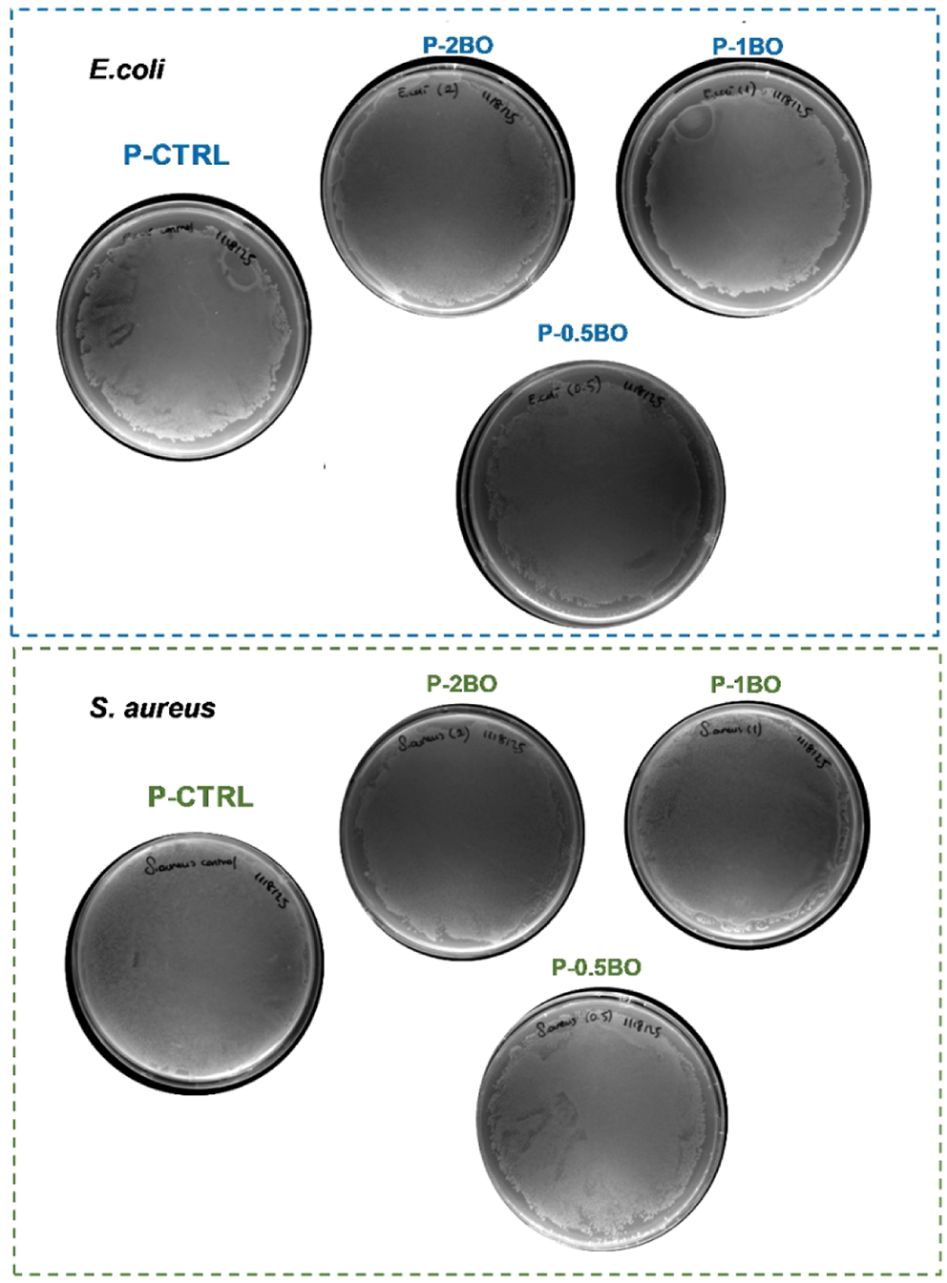
Images of 1:1000 diluted bacterial cultures containing the film samples after overnight growth on LB agar plates.

The observed differences in bacterial susceptibility also underscore the relevance of targeting the structural vulnerabilities of specific bacterial groups. Gram-positive bacteria, due to their relatively simple envelope architecture and absence of an outer membrane, are more prone to disruption by surface-active or charge-bearing polymeric materials. In contrast, the outer membrane of Gram-negative bacteria not only limits penetration but also harbors efflux pumps and other resistance mechanisms that can further diminish the efficacy of antimicrobial agents.^74^

From an application standpoint, the enhanced antibacterial performance of the 2BO and 0.5BO films against both bacterial species, and especially against *S. aureus*, suggests potential utility of these materials in biomedical and especially packaging applications where prevention of Gram-positive bacterial contamination is critical. However, the reduced activity against *E. coli* indicates that additional modifications, such as the incorporation of metallic nanoparticles (e.g., Ag or ZnO), natural antimicrobial agents (e.g., essential oils, chitosan), or surface functionalization strategies, may be required to achieve broad-spectrum antibacterial activity.^72^ Overall, the present findings contribute to the growing body of evidence that Gram-positive bacteria are generally more susceptible to polymer-based antimicrobial systems, while Gram-negative bacteria often require more aggressive or synergistic strategies to overcome their complex defense mechanisms. Further studies focusing on the molecular interactions between these polymers and bacterial cell envelopes, as well as long-term stability and cytocompatibility, will provide valuable insights for the development of next-generation antibacterial polymer composites.

Moreover, ICP-OES analysis was performed in order to determine the boron oxide leaching from the PLA film to clarify the effect of possible BO leaching on the antimicrobial activity. According to obtained results, no detectable boron release (0.000 mg L^–1^) was observed for the film with the lowest BO content (P-0.5BO). In addition, in the case of the highest BO-loaded sample (P-2BO), the boron concentration detected in the leachates was extremely low (0.0018 mg L^–1^), indicating that BO concentration leached from P-BO films was far below the concentration affecting antimicrobial activity. Overall, ICP-OES results confirmed that the BO species were strongly immobilized within the PLA matrix with high chemical stability and leaching resistance.

## 4 Conclusion

In the present study, different loading level BO reinforced PLA/PEG composite films were produced by the solution casting technique. The structures of the obtained BO containing films were determined by using FTIR analysis. The BO-containing films’ FTIR spectra show that BO2 was successfully integrated into the polymer matrix, resulting in structural modifications. The impact of loading level on the composite films was examined by thermal, mechanical, water vapor permeability, morphological, and antibacterial activity. According to TGA analyses, the addition of BO increased the thermal stability of the PLA/PEG matrix, showing a weight loss of 10% at approximately 338 °C (±2). In film samples containing 0.5%, 0.7%, and 1%, BO increased tensile strength by acting as a reinforcing agent. The greatest increase in tensile strength occurred at approximately 72% at the 0.5% BO content compared to the control sample. However, increases in BO content reduced the improvement in tensile strength. The plasticized PLA control film has exhibited a relatively high WVTR, and adding BO to the matrix at low concentrations (0.5 and 0.7) resulted in a significant reduction in WVTR. Based on SEM analysis, there are no visible interfacial voids, and the cross-section is continuous and generally compact at low BO loadings, indicating good wetting and adhesion between BO and the PLA/PEG matrix. When the BO loading is increased, the top surface displays noticeable ridges and clusters on a few microns scale, which are consistent with local BO enrichment, and the cross-section displays discrete filler aggregates embedded in the matrix. In the agar-based disk diffusion assay, zones of inhibition increased with BO loading (P-0.5BO < P-1BO < P-2BO), indicating a dose-dependent increase in antibacterial activity. In contrast, P-0.5BO reduced growth more than P-2BO in liquid culture. Collectively, the data showed that the addition of BO enhanced the antibacterial performance of the films, and the magnitude of the dose effect depended on the experimental conditions. Furthermore, BO incorporated into PLA/PEG films exhibited greater antimicrobial activity against *S. aureus* compared to *E. coli*. From a sustainability perspective, the biodegradation behavior of PLA is expected to be preserved in the presence of low amounts of boron-containing additives. Since PLA degrades mainly via hydrolysis followed by microbial assimilation under composting conditions, and the inorganic boron phase does not biodegrade, the overall degradation mechanism remains dominated by the polymer matrix.^75–78^ The boron-containing residue is therefore expected to persist as an inorganic component after matrix degradation without preventing compostability. When all the results are evaluated together, it is suggested that the developed films can be potentially used in biomedical and especially packaging applications where the prevention of Gram-positive bacterial contamination is critical due to the BO content.
